# Extra Fructose in the Growth Medium Fuels Lipogenesis of Adipocytes

**DOI:** 10.1155/2014/647034

**Published:** 2014-02-19

**Authors:** Armin Robubi, Klaus R. Huber, Walter Krugluger

**Affiliations:** Department of Laboratory Medicine, Donauspital, SMZ Ost, Langobardenstraße 122, 1220 Vienna, Austria

## Abstract

Fructose in excessive amounts exerts negative effects on insulin sensitivity, blood pressure, and liver metabolism. These adverse outcomes were attributed to its disturbances of key metabolic pathways in the liver. Recently, possible consequences of high fructose levels directly on adipocytes *in vivo* have been considered. We have cultured adipocytes in growth media containing 1 g/L fructose additionally to glucose and monitored the cells fate. Cells developed lipid vesicles much earlier with fructose and showed altered kinetics of the expression of mRNAs involved in lipogenesis and hexose uptake. Adiponectin secretion, too, peaked earlier in fructose containing media than in media with glucose only. From these data it can be speculated that similar effects of fructose containing diets happen *in vivo* also. Apart from toxic action on liver cells, adipocytes might be stimulated to take up extra fructose and generate new lipid vesicles, further dysregulating energy homeostasis.

## 1. Introduction

The last thirty years have seen a global epidemiological rise in obesity. Currently, the number of obese people exceeds the number of malnourished people by thirty percent. The increase is linked strongly with rapid rises for a number of serious conditions such as type-2-diabetes, hypertension, cardiovascular disease, dyslipidemia, sleep apnea, and some kinds of cancer [1, 2].

The obesity epidemic was accompanied among many other factors by a steep rise in the consumption of sucrose and high-fructose corn syrup through sugar sweetened beverages and other products. In fact, even adults trying to lose weight take in more than 700 mL of sugary drinks a day on average [3]. A number of studies have implicated a role of high-fructose consumption in hypertension, hyperuricemia, hyperinsulinemia, dyslipidemia, hepatic insulin resistance, nonalcoholic fatty liver disease (NAFLA), leptin resistance, type-2-diabetes, and obesity (summarized by [4]).

Most studies on adverse effects of fructose investigate hepatic effects of fructose. In the current study we aimed to elucidate if adipocytes in culture tolerate high amounts of fructose and if fructose may promote obesity by acting on adipocytes directly as has been suggested previously based on human studies [5]. For this purpose we cultured primary human preadipocytes in growth medium containing usual amounts of glucose (1 g/L) with added fructose (1 g/L). Fructose treated cells were screened for expression levels of genes involved in hexose uptake, insulin resistance, and *de novo* lipogenesis. Additionally, we monitored the secretion of adiponectin, a key cytokine secreted form adipocytes which affects insulin resistance and inflammation.

## 2. Materials and Methods

### 2.1. Cell Culture

Primary human white preadipocytes (HWP-c) were obtained from PromoCell (Heidelberg, Germany). Preadipocytes were grown in preadipocytes growth medium containing 5% fetal bovine serum, 4 *μ*L/mL endothelial cell growth supplement, 10 ng/mL epidermal growth factor (EGF), and 1 *μ*g/mL hydrocortisone (PromoCell) at 37°C in humidified air containing 5% CO_2_. All cell media contained 1 g/L of glucose. Fructose when added was used at a 1 g/L concentration. Cells were inspected visually once a day and fresh growth medium was added every third or fourth day. An aliquot of the depleted medium was stored at −80°C and later used to measure fructose, glucose, lactate, and adiponectin.

### 2.2. Differentiation

Twenty-four hours after cells reached 100% confluence, cells were differentiated by 72 hours of treatment in preadipocyte differentiation medium containing no serum, 8 *μ*g/mL d-biotin, 0.5 *μ*g/mL insulin, 400 ng/mL dexamethasone, 44 *μ*g/mL 3-isobutyl-1-methylxanthin (IBMX), 9 ng/mL L-thyroxine, and 3 *μ*g/mL ciglitazone (PromoCell) with or without 1 g/L fructose. Subsequently, the media were replaced by adipocyte nutrition medium (PromoCell) containing 3% FBS, 8 *μ*g/mL d-biotin, 0.5 *μ*g/mL insulin, and 400 ng/mL dexamethasone, again with or without added fructose and cell cultures were inspected visually once a day.

Glucose and lactate concentrations were measured using standard clinical laboratory methods. Fructose concentrations were measured using a commercial kit (EFRU-100, Bioassay Systems, Hayward, USA). Adiponectin levels were measured with a commercial ELISA kit (KA0017, Abnova, Taipei, Taiwan).

### 2.3. RT-PCR

Cells were lysed in Trizol Reagent (Life Technologies, Darmstadt, Germany) and RNA was purified according to the manufacturer's instructions. To this end, aliquot flasks of differentiating/differentiated adipocytes were rinsed with Trizol for immediate mRNA preservation and extraction after the culture medium was collected (and frozen at −70°C for later processing). mRNA was reverse transcribed directly and the cDNA was stored at −70°C. This protocol ensured the highest possible quality of mRNA, preserving the mRNA within seconds from nuclease digest.

Random primers, deoxynucleotide triphosphates, protector RNAase inhibitor, and reverse transcriptase were obtained from Roche (Basel, Switzerland). RT-PCR was performed using Roche FastStart DNA Master HybProbe (Roche) and Taqman primers and probes for fatty acid synthase (FASN; Hs01005622), glycerol-3-phosphate acyltransferase (GPAM; Hs0157368), solute carrier family 2, member 1 (GLUT1, also known as SLC2A1; Hs0089268), Solute Carrier Family 2, member 4 (GLUT4, also known as SLC2A4; Hs00168966), Solute Carrier Family 2, member 5 (GLUT5, also known as SLC2A5; Hs0016172), adiponectin (Hs00605917), leptin (Hs00174877), and interleukin 6 (IL6; Hs00985639) obtained from Life Technologies. RT-PCR was performed on an ABI Prims 7000 detection system (Life Technologies). FAM fluorescence was used as readout. The amplification blots were checked visually and the baseline was set manually.

### 2.4. Statistical Analysis

All experiments were performed twice. Apart from preadipocytes were *t*-test analysis (SPSS V19, IBM, Armonk, NY, USA) was sought, the figures showing the time courses depict the raw data of one representative experiment.

## 3. Results

### 3.1. Preadipocytes

The first aim of this study was to realize whether or not human adipocytes can tolerate the presence of fructose in the culture medium. Cells were cultured in preadipocyte growth medium with or without added fructose (1 g/L) for up to two weeks. Cells (with or without added fructose) reached confluence simultaneously, could be replated after trypsin incubation, and were indistinguishable morphologically in both culture media.

When it was obvious that these cells showed tolerance to the high-fructose level in the medium, differing biochemical mechanisms reflecting addition of the two hexoses were sought by analyzing expression of various genes for the uptake, utilization, and effects of fructose. To this end, mRNA levels of target genes were compared to the amount of actin mRNA and/or GAPDH mRNA. Indeed, prolonged exposure of the cells to fructose showed differing patterns of mRNA expression compared to the cells incubated with glucose only.

The amount of mRNA for the cellular receptor for fructose (GLUT5) showed a trend towards reduction after incubation of the cells with fructose for more than a week (data not shown).

Fructokinase mRNA was expressed in such minute amounts in these cells that no meaningful analysis was possible (low levels were observable in differentiated adipocytes, though). These preadipocytes did not produce adiponectin or leptin, no matter which hexose was present in the medium. These cells (and much more so differentiated adipocytes) do show differing reactions towards incubation with fructose because their levels of IL6 mRNA showed a trend towards twice that of cells incubated with glucose only after more than a week (*n* = 7, two-sided *t*-test, *P* = 0.057; data not shown).

### 3.2. Adipocytes

Four days after the cells were incubated in adipocyte nutrition medium, cells were inspected visually, media were collected, and mRNA was extracted daily. The first hints of fructose effects were found by observing the cells in the microscope. Fat droplets within the cells developed quicker and to a greater extend in cells incubated with both sugars (data not shown) than with glucose only.

We were interested in the utilization of the different hexoses by the cells and measured glucose consumption (and lactate production) with an automated analyzer and fructose consumption with a biochemical assay. Because media were changed at 3 to 5 days intervals, we calculated sugar ingestion by dividing the total decrease of the respective sugar in the media by the number of days the cells were in contact with the respective media. By this, hexose utilization per day could be inferred and related to lactate production by the cells: During 14 days, lactate production was comparable in fructose containing media (varying between 600 nmol and 1200 nmol per day per 10 square centimeter well) and glucose only containing media (500–1000 nmol/day/10 cm^2^ well). Total sugar consumption paralleled lactate production. Combined consumption of fructose and glucose in the fructose containing medium was at comparable levels as glucose consumption in medium containing glucose only. Thus, cells in media containing both hexoses utilized amounts of glucose and fructose that about summoned the utilization of glucose in media with glucose only (Table 1). These data imply that same amounts of cells (all wells were confluent) utilize same amounts of energy independent of the source.

Furthermore, quantitative data about different utilization of fructose versus glucose were retrieved by mRNA expression analysis. The amounts of actin mRNA served as quantitation standard for the other genes monitored. GAPDH mRNA levels were tested as standards also; both yielded same results.

**Figure 1:**
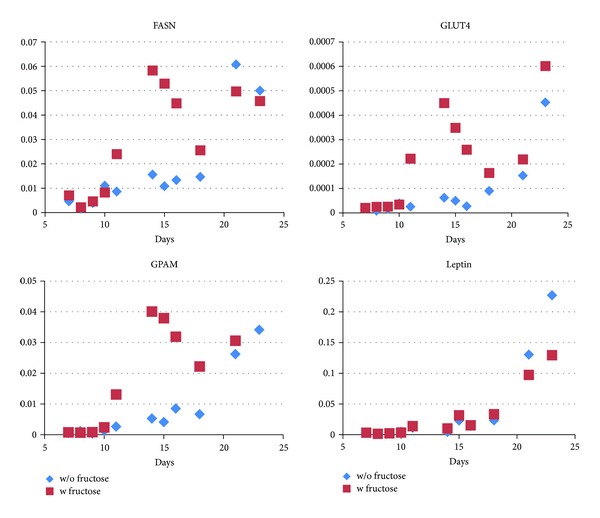
Fructose transiently induces genes involved in lipogenesis during adipocyte maturation. Expression of important lipogenic genes is shown in the presence (red squares) and in the absence of fructose (blue diamonds). *y*-axis: relative expression compared to expression of actin mRNA. FASN and GPAM are strongly induced between day ten and sixteen. The insulin-dependent glucose transporter GLUT4 follows the same course. No significant differences are seen for leptin expression. Each symbol depicts one qPCR measurement per dish in a representative experiment.

**Figure 2:**
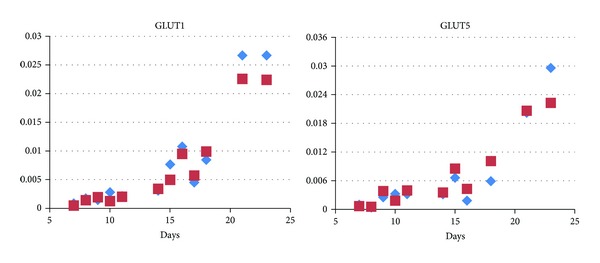
Expression of the glucose transporter GLUT1 and the fructose transporter GLUT5. Both transport proteins exhibit a steep rise during maturation. However, no difference is seen in the absence (blue diamonds) and in the presence (red squares) of fructose. *y*-axis: relative expression compared to expression of actin mRNA. Each symbol depicts one qPCR measurement per dish in a representative experiment.

First, the quantity of the respective sugar transporter mRNA was compared. Cells with glucose only in the growth medium showed slightly increasing GLUT4 (insulin-dependent glucose receptor) levels over two weeks with a marked rise after 14 days in the medium. In contrast, the parallel presence of equimolar amounts of fructose in the medium (1 g/L) led to a sharp increase after 7 days (20 times higher than at day one) that dropped after 14 days to the levels seen in glucose only containing media and raised again to higher levels in these cells at the end of the experiment (day 22 in adipocyte nutrition medium) (Figure 1). GLUT5 (the major fructose transporter) levels and the insulin independent glucose receptor GLUT1, however, increased steadily up to a similar extent in both media (Figure 2).

The kinetics of GPAM and FASN mRNA levels were concordant with the GLUT4 transporter mRNA levels (and the faster development of lipid droplets) in fructose containing media. Both genes are involved in *de novo* lipogenesis. Whereas these levels increased only slightly over the first 14 days in glucose medium and raised sharply, thereafter, in fructose medium the time course was quite different: already one week after the cells had been differentiated, a sharp increase of GPAM and FASN levels could be observed, substantiating the observed faster development of lipid droplets. The expression of these genes reached the maximum levels ten days earlier than cells incubated with glucose only. This peak decreased thereafter continuously until the end of the experiment. No such dynamics was observed with leptin mRNA levels (Figure 1).

We were interested whether or not this decline reflected dedifferentiation or apoptosis of the cells induced by the high levels of fructose in the medium. Expression levels of delta-like 1 homolog (DLK1) (a marker for preadipocytes) measured on all time points of differentiation and maintenance indicated no dedifferentiation to preadipocytes. Similarly, apoptosis as corroborated by increasing levels of CASP8 and FADD-Like Apoptosis Regulator (CFLAR) could not be seen (data not shown). We suppose, therefore, that the cells had reached the end of their maturation and final size in the adipocyte nutrition medium much earlier with fructose.

**Figure 3:**
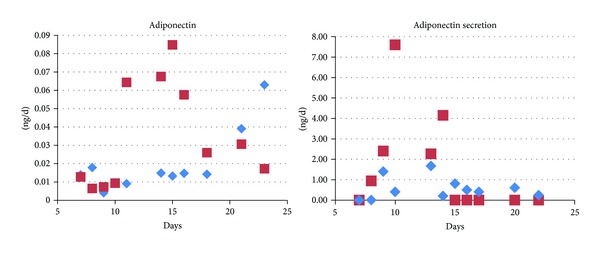
Adiponectin expression and secretion in response to fructose. Fructose (red squares) induces adiponectin expression transiently. The time kinetics resembles that of the lipogenic genes (Figure 1). The rise is not limited to the adiponectin transcript as cells also excrete more adiponectin during maturation when treated with fructose. Adiponectin secretion ceased once maturation was completed, irrespective of fructose in the medium. Each symbol depicts one qPCR/ELISA measurement per dish in a representative experiment.

The differentiated adipocytes expressed adiponectin and excreted it into the medium. In general, expression levels were rather low. However, in the presence of fructose, again a strong rise of the adiponectin transcript, as well as high adiponectin excretion, was observed transiently during maturation (Figure 3).

Finally, we monitored IL6 mRNA levels. The time courses in both media were comparable, showing slightly increasing levels that diminished towards the end of the experiment (data not shown).

In summary, our data provide evidence of substantial effects of fructose on adipocyte fate in cell culture.

## 4. Discussion

Obesity is one of the most pressing issues of the entire health care systems, even in developing countries. The rapid rise of obesity is accompanied—among many other factors—by rises in fructose consumption. Consumption of sugar sweetened beverages that contain high amounts of fructose alone has risen by more than 100% in the United States between 1980 and 2006 [6].

Fructose is metabolized mainly by the liver. Readily available fructose from sugar sweetened drinks and highly processed foods is quickly taken up and exhibits a strong first-pass effect [7]. The liver eliminates a high portion of the fructose from the blood stream during its first passage. This is achieved mainly by the action of ketohexokinase which rapidly converts fructose to fructose-1-phosphate in the hepatocytes [8]. Most of the deleterious effects of fructose are attributed to this rapid conversion and the subsequent effects of fructose-1-phosphate and its secondary metabolites [7]. Adipocytes do express the fructose transporter GLUT5—the major specific fructose transporter [9]—but they normally express only minute amounts of ketohexokinase. Fructose may anyhow be metabolized in these cells after being phosphorylated by hexokinase to fructose-6-phosphate—albeit much slower compared to hepatocytes.

Ketohexokinase knockout mice are protected from the harmful metabolic effects of fructose, whereas in tissue specific knockouts, which express ketohexokinase only in the liver and no other tissue, the deleterious effects are exacerbated [10]. A high-fructose diet has also been shown to inhibit insulin-induced repression of lipolysis in adipocytes [5]. The authors were not able to elucidate the exact mechanisms of this effect, but they speculated that a fraction of the fructose may reach systemic circulation and act directly on adipose tissue. In this study, we could show that this indeed is what is probably happening *in vivo* and, moreover, fructose induces adipocytes to a rapid rise of fat content by inducing several key metabolic genes. It must be pointed out that we added rather high amounts of fructose. However, further experiments with primary adipocyte cultures from bariatric surgery patients with 0.1 g/L fructose together with 1 g/L glucose did show similar effects (to be published soon). It might be argued that—instead of adding fructose—higher amounts of glucose might have similar effects. However, our primary aim was the confirmation that adipocytes can tolerate high levels of fructose that may be reached *in vivo* by bolus administration of sweetened drinks.

Fructose in our cultures drives up expression of GLUT4, the major insulin-dependent glucose transporter in adipocytes [11]. We did not observe an increase in glucose uptake in the presence of fructose but this may be due to the constant insulin levels in our media. *In vivo*—particularly under the condition of the metabolic syndrome and insulin resistance—the situation may be different and weight gain and lipogenesis might also be in part driven by excessive uptake of glucose. Interestingly, we saw also an upregulation of adiponectin in cells treated with fructose. Adiponectin is an abundant anti-inflammatory cytokine which is secreted by adipose tissue and has been implicated to play a major role in insulin sensitivity and obesity [12]. However, it should be noted that the overall expression levels of adiponectin in our cells is much lower than in adipose tissue where the adiponectin transcript exhibits about the same abundance as actin does (own data). Most likely, our cell culture system fails to emulate the physiological conditions with regard to adiponectin, possibly due to lack of specific cytokines and hormones.

We chose FASN—a key enzyme in the synthesis of fatty acids from acetyl-CoA [13]—and GPAM—which catalyzes the first rate limiting step in the synthesis of glycerolipids [14]—mRNA expression to monitor lipid synthesis in the cells. Indeed, FASN and GPAM mRNA levels together with the generation of lipid vesicles raised much faster in adipocytes incubated with fructose and glucose than in cells incubated with glucose only. Ten days before cells with glucose reached their maximum values, cells with fructose and glucose showed the apex of expression levels of these genes.

Finally, we monitored IL6 mRNA levels, a proinflammatory cytokine that might play a role in obesity and metabolic syndrome [12]. Their time courses in both media were comparable, showing slightly increasing levels that diminished towards the end of the experiment.

Importantly, all the effects of added fructose observed in our *in vitro* culture system must be considered happening under medium conditions that provide cells with plenty of nutrients already (1 g/L glucose) of which only minor amounts are metabolized between medium renewals. From these data it can be speculated that similar effects of fructose containing diets happen *in vivo* also. Apart from toxic action on liver cells, adipocytes might be stimulated to take up extra fructose and generate new lipid vesicles, further dysregulating energy homeostasis.

## Figures and Tables

**Table 1:** Fructose is readily taken up by adipocytes. The amount (mean) refers to one confluent 10 cm^2^ well (combined data of 14 days). Control: glucose only; fructose: glucose plus fructose in the media.

	nmol/d/10 cm^2^	Std. dev.
Glucose uptake control	660	520
Glucose uptake + fructose	420	300
Fructose uptake control	—	—
Fructose uptake + fructose	530	430
Lactate secretion control	1630	370
Lactate secretion + fructose	1440	480
